# Gender Differences in Primary Care Physicians’ Electronic Health Record Use over Time: an Observational Study

**DOI:** 10.1007/s11606-022-07837-2

**Published:** 2022-12-06

**Authors:** Adam Rule, Christina M. Shafer, Mark A. Micek, Jeffrey J. Baltus, Christine A. Sinsky, Brian G. Arndt

**Affiliations:** 1 Information School, University of Wisconsin–Madison, Madison, WI USA; 2 Department of Medicine, University of Wisconsin–Madison, Madison, WI USA; 3 Present Address: Independent Consultant, Madison, WI USA; 4 Department of Family Medicine and Community Health, University of Wisconsin–Madison, Madison, WI USA; 5 Professional Satisfaction and Practice Sustainability, American Medical Association, Chicago, IL USA

## INTRODUCTION

Female physicians spend more time interacting with electronic health records (EHRs) and experience higher rates of burnout than male physicians.^1–3^ This study sought to assess whether the gender gap in EHR use has changed over time for primary care physicians (PCPs), who use EHRs more than physicians in other specialties.^4^

## METHODS

This retrospective cross-sectional study was approved by the University of Wisconsin’s institutional review board. We collected monthly EHR usage (Epic Systems; Verona, WI), scheduling, panel, and demographic data for all PCPs who practiced at UW Health—a large academic medical center serving over 290,000 medically homed patients—from April 2019 to March 2022. We calculated five measures of monthly EHR use for each physician and normalized each measure per 8 hours of scheduled clinic: total EHR time (EHR_8_), time in notes (Notes_8_), time in inbox (Inbox_8_), time in orders (Orders_8_), and time outside scheduled clinic hours or “Work Outside of Work” (WOW_8_).^5^

We performed unadjusted comparisons of physician demographics, workload, and EHR use with *χ*^2^ tests for count data and Mann-Whitney *U* tests for continuous variables. We used multivariable linear regression to perform adjusted comparisons of EHR use by gender. We modeled gender differences in EHR use during the last year of the study (April 2021–March 2022), controlling for physician specialty, duration of employment, panel size, monthly visit volume, normalized visit volume (Visit_8_), and combined normalized volume of four labor-intensive inbox message types: laboratory results management, medication management, telephone calls, and patient portal messages (Msg_8_). We modeled longitudinal changes in the gender gap by revising the above models to include data from all three study years (April 2019–March 2022), adding a term for the interaction between physician gender and study year, and controlling for year and month to account for potential seasonality. Each model used random effects to account for longitudinal correlation in individual physician’s EHR use. Data from March to May 2020 were excluded from analysis due to the transitory changes in EHR use early in the COVID-19 pandemic.^6^ Vendor-provided EHR usage data were missing for February and April 2021, so these months were likewise excluded.

## RESULTS

One-hundred-and-sixty PCPs were included in this study (Table 1). In unadjusted comparisons across April 2021–March 2022, female physicians had higher EHR_8_ (mean, 7.0 versus 5.3 h; *p*<.001), Note_8_ (2.5 versus 1.4 h; *p*<.001), Inbox_8_ (1.3 versus 1.0 h; *p*=0.01), and WOW_8_ (3.1 versus 2.0 h; *p*<.001), but not Orders_8_ (1.0 versus 0.9 h; *p*=0.27). In model-adjusted comparisons over the same period, female physicians had higher EHR_8_ (1.5 h, 95%CI 0.7–2.4 h; *p*<.001), Note_8_ (0.9 h, 95%CI 0.5–1.4 h; *p*<.001), Inbox_8_ (0.2 h, 95%CI 0.1–0.4 h; *p*=.01), and WOW_8_ (1.1 h, 95%CI 0.4–1.8 h; *p*=.002), but not Orders_8_ (0.1 h, 95%CI 0.0–0.2 h; *p*=0.15).

**Table 1:** Observed Physician Demographics and Workload

Characteristic	Physicians, *N* (%)			
	All (*N*=160)	Male (*N*=52)	Female (*N*=108)	*p* value
Specialty				
Family medicine	80 (50.0)	30 (57.7)	50 (46.3)	0.22^a^
General internal medicine	54 (33.8)	17 (32.7)	37 (34.3)	
Pediatrics	26 (16.3)	5 (9.6)	21 (19.4)	
Duration of employment				
<5 years	21 (13.1)	4 (7.7)	17 (15.7)	0.46^a^
5–10 years	47 (29.4)	18 (34.6)	29 (26.9)	
10–15 years	31 (19.4)	11 (21.2)	20 (18.5)	
15–20 years	22 (13.8)	5 (9.6)	17 (15.7)	
>20 years	39 (24.4)	14 (26.9)	25 (23.1)	
Workload^c^, mean (SD)				
Clinical FTE	0.64 (0.21)	0.68 (0.25)	0.63 (0.19)	0.04^b^
Panel size^d^	1432 (525)	1564 (587)	1369 (482)	0.02^b^
Monthly visits	139.3 (44.5)	148.7 (51.0)	134.7 (40.4)	0.05^b^
Visit_8_	15.1 (2.4)	14.8 (2.3)	15.3 (2.4)	0.08^b^
Msg_8_	52.1 (20.8)	53.4 (17.9)	51.5 (22.1)	0.26^b^

^a^*P* value from *χ*^2^ test. ^b^*P* value from Mann-Whitney *U* test. ^c^Workload average across April 2021 to March 2022. ^d^Active panel weighted by patient age, patient gender, payor, and relative value units associated with patient care

The gender gap in EHR use increased from 2019 to 2022 (Table 2). In model-adjusted comparisons, this gap increased by 8.3 min/year for EHR_8_ (95%CI 4.6–11.9 min/year; *p*<.001), 6.1 min/year for Note_8_ (95%CI 4.1–8.0 min/year; *p*<.001), 3.3 min/year for Inbox_8_ (95%CI 2.2–4.4 min/year; *p*<.001), and 6.2 min/year for WOW_8_ (95%CI 2.7–9.6 min/year; *p*<.001).

**Table 2:** Observed Duration of EHR Use by Gender, Modeled Change in Gender Gap over Time

	Year 1^a^, mean hours (SD)		Year 2^b^, mean hours (SD)		Year 3^c^, mean hours (SD)		Change in gap, female to male, minutes per year^d^	
	Male	Female	Male	Female	Male	Female	Est. (95%CI)	*p*
EHR_8_	5.0 (2.1)	6.4 (2.5)	5.1 (2.5)	6.5 (2.5)	5.3 (2.4)	7.0 (2.8)	8.3 (4.6 to 11.9)	<.001
Note_8_	1.4 (1.0)	2.3 (1.3)	1.4 (1.1)	2.3 (1.3)	1.4 (1.1)	2.5 (1.4)	6.1 (4.1 to 8.0)	<.001
Inbox_8_	0.8 (0.4)	1.0 (0.4)	1.0 (0.5)	1.2 (0.5)	1.0 (0.5)	1.3 (0.7)	3.3 (2.2 to 4.4)	<.001
Orders_8_	0.6 (0.2)	0.7 (0.3)	0.7 (0.3)	0.8 (0.3)	0.9 (0.4)	1.0 (0.4)	−0.3 (−1.0 to 0.4)	0.44
WOW_8_	1.7 (1.7)	2.6 (1.7)	1.9 (2.2)	2.8 (1.8)	2.0 (2.0)	3.1 (2.2)	6.2 (2.7 to 9.6)	<.001

^a^April 2019 to March 2020. ^b^April 2020 to March 2021. ^c^April 2021 to March 2022. ^d^Estimated with linear regression model with controls for provider, specialty, duration of employment, weighted active panel, monthly visit volume, normalized visit volume, normalized message volume, month, and year

## DISCUSSION

This cross-sectional study found female PCPs spend more time interacting with EHRs than male PCPs. The gaps observed in this study are larger than those reported in prior work,^1,2^ which may be due to differences in health systems, time periods, or specialties observed. The gender gap in EHR use is growing, particularly for note writing, inbox management, and time outside scheduled clinical hours (WOW). The gap in WOW is particularly concerning given the association between outside-hours EHR use and physician burnout.^7^ It is critical that health systems explore root causes behind these differences and develop solutions to address them considering the higher burnout among female physicians.^3^ Possible solutions include additional documentation and inbox support, reduced panel size, and/or reduced patient contact time.

This study observed PCPs’ ambulatory use of a single EHR at a single academic health center. As an observational study, it could not determine the cause of observed differences, whether they might be attributed to asynchronous work patterns, patient population, or other systemic factors. Future work might examine the gender gap in EHR use in other domains and the impact of targeted interventions to reduce EHR burden for female physicians.

## References

[1] **Rotenstein LS, Fong AS, Jeffery MM, Sinsky CA, Goldstein R, Williams B, Melnick ER.** Gender differences in time spent on documentation and the electronic health record in a large ambulatory network. JAMA Netw Open. 2022;5(3):e223935-. 10.1001/jamanetworkopen.2022.3935 PMC8948526 35323954

[2] **Rittenberg E, Liebman JB, Rexrode KM.** Primary care physician gender and electronic health record workload. J Gen Intern Med. 2022;6:1-7. 10.1007/s11606-021-07298-z PMC9550938 34993875

[3] **West CP, Dyrbye LN, Shanafelt TD.** Physician burnout: contributors, consequences and solutions. J Intern Med. 2018;283(6):516-29. 10.1111/joim.12752 29505159

[4] **Rotenstein LS, Holmgren AJ, Downing NL, Bates DW.** Differences in total and after-hours electronic health record time across ambulatory specialties. JAMA Intern Med. 2021;181(6):863-5. 10.1001/jamainternmed.2021.0256 PMC7985815 33749732

[5] **Sinsky CA, Rule A, Cohen G, et al.** Metrics for assessing physician activity using electronic health record log data. J Am Med Inform Assoc. 2020;27(4):639-43. 10.1093/jamia/ocz223 PMC7075531 32027360

[6] **Holmgren AJ, Downing NL, Tang M, Sharp C, Longhurst C, Huckman RS.** Assessing the impact of the COVID-19 pandemic on clinician ambulatory electronic health record use. J Am Med Inform Assoc. 2022;29(3):453-60. 10.1093/jamia/ocab268 PMC8689796 34888680

[7] **Adler-Milstein J, Zhao W, Willard-Grace R, Knox M, Grumbach K.** Electronic health records and burnout: time spent on the electronic health record after hours and message volume associated with exhaustion but not with cynicism among primary care clinicians. J Am Med Inform Assoc. 2020;27(4):531-8. 10.1093/jamia/ocz220 PMC7647261 32016375

